# Multiple Protein Profiler 1.0 (MPP): A Webserver for Predicting and Visualizing Physiochemical Properties of Proteins at the Proteome Level

**DOI:** 10.1007/s10930-024-10214-z

**Published:** 2024-07-09

**Authors:** Gustavo Sganzerla Martinez, Mansi Dutt, Anuj Kumar, David J Kelvin

**Affiliations:** 1 Department of Microbiology and Immunology, Dalhousie University, Halifax, NS B3H4H7 Canada; 2 Department of Pediatrics, Izaak Walton Killam (IWK) Health Center, Canadian Center for Vaccinology (CCfV), Halifax, NS B3H4H7 Canada; 3 BioForge Canada Limited, Halifax, NS Canada

**Keywords:** Proteome, Proteins, Physicochemical, Webserver, Bioinformatics

## Abstract

Determining the physicochemical properties of a protein can reveal important insights in their structure, biological functions, stability, and interactions with other molecules. Although tools for computing properties of proteins already existed, we could not find a comprehensive tool that enables the calculations of multiple properties for multiple input proteins on the proteome level at once. Facing this limitation, we developed Multiple Protein Profiler (MPP) 1.0 as an integrated tool that allows the profiling of 12 individual properties of multiple proteins in a significant manner. MPP provides a tabular and graphic visualization of properties of multiple proteins. The tool is freely accessible at https://mproteinprofiler.microbiologyandimmunology.dal.ca/.

**Supplementary Information:**

The online version contains supplementary material available at 10.1007/s10930-024-10214-z.

## Introduction

Proteins are the ubiquitous biological macromolecules that play principal roles in living organisms. These large and complex molecules have been well studied for their involvement in the structure, function, and regulation of tissues and organs [1]. In addition, these molecules are important structural components that perform a plethora of biological functions, from replicating DNA and transporting/storing molecules to catalyzing metabolic pathways and much more [2]. Twenty different amino acids significantly contribute to the formation of proteins in the body. Antibodies, enzymes, hormones, messengers, structural components, and transport/storage molecules are among the popular examples of proteins. At the structural level, proteins can be defined by their primary, secondary, tertiary, and quaternary structures [3, 4]. Physiochemical properties of the proteins play a vital role in determining their structures, biological functions, stability, and molecular interactions with other micro- and macromolecules [5]. At the sequence level, a set of properties including molecular weight, theoretical isoelectric point (pI), estimated half-life, instability index, amino acid composition, atomic composition, aliphatic index, and grand average of hydropathicity (GRAVY) index, are defined as the popular physiochemical properties of proteins. These properties are crucial to the understanding and annotation of the complex biological processes [6–8]. So far, a number of tools and databases are available in the public domain for predicting the physiochemical properties of proteins, including ExPASy ProtParam (https://web.expasy.org/protparam/); AAindex (https://www.genome.jp/aaindex/); PeptideMass (https://web.expasy.org/peptide_mass/); and Biopython (https://biopython.org/). Recently, Chauhan et al. [9] developed the GUD-VE, a graphical user interface (GUI) tool for visualizing the physicochemical properties of proteins. However, no web server is available for the prediction of physiochemical properties of a bulk of proteins or whole proteome. We therefore developed a user-friendly webtool, the Multiple Protein Profiler 1.0 (MPP) (http://mproteinprofiler.microbiologyandimmunology.dal.ca/), to simultaneously predict the physiochemical properties of either a bulk of proteins or whole proteomes. This webserver also facilitates end users in rendering predicted results in the form of attractive, ready-to-publish graphs. The MPP web server is, to our knowledge, the first of its kind. We anticipate that the MPP will be useful for the scientific community to predict and annotate the biological functions of the proteins.

## Development

**Fig. 1:**
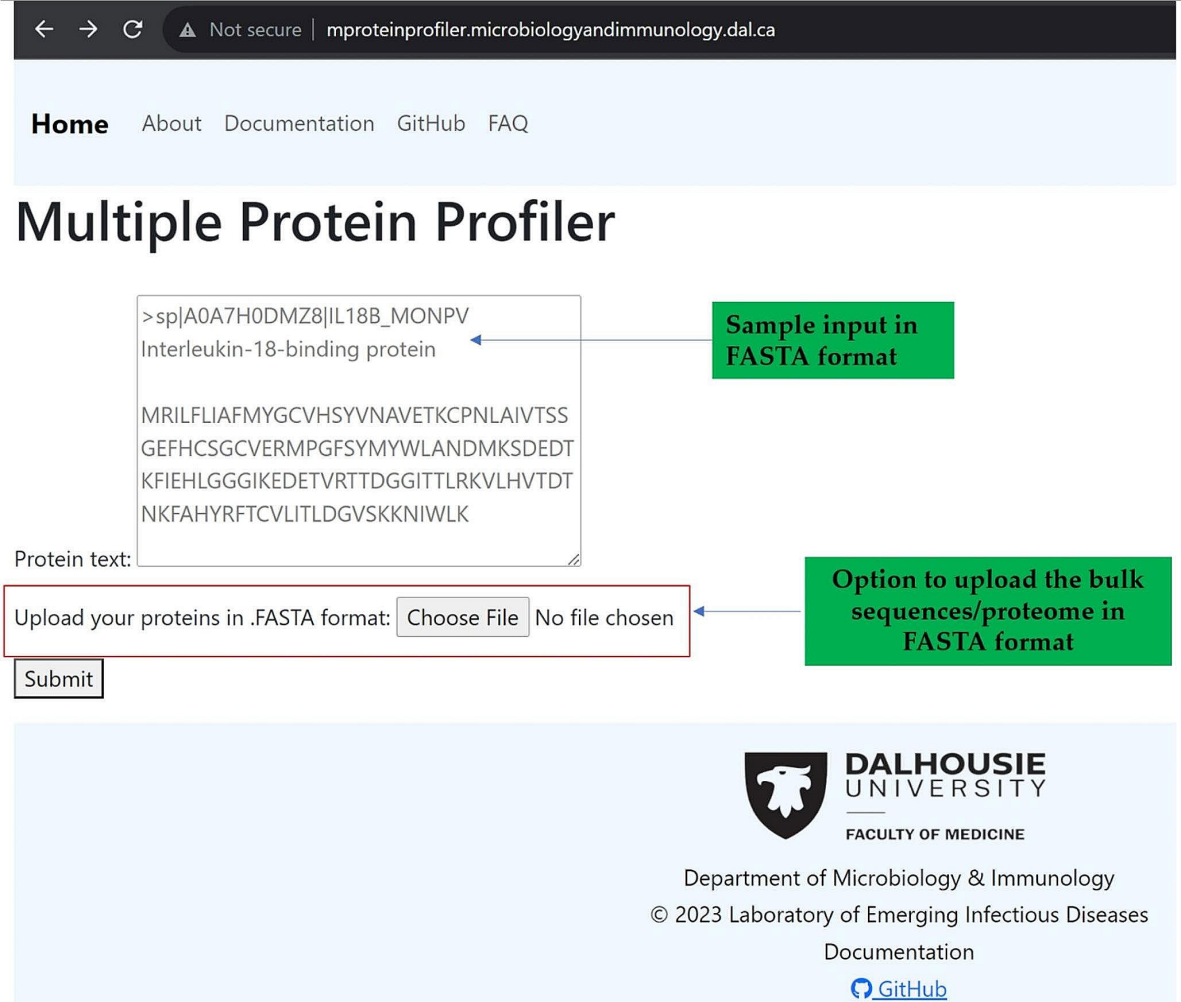
Homepage of the Multiple protein Profiler with the options of sample input and upload files in fasta format

The Multiple Protein Profiler (MPP) tool was developed using Python version 3.10.5 in the backend and Hypertext Markup Language (HTML) 5 in the frontend; MPP is implemented as a web application using the Django framework. Since MPP is not platform/browser dependent, it is able to run in both desktop and mobile versions of Google Chrome, Mozilla Firefox, Apple’s Safari, Opera, and Microsoft Edge. No database is required for MPP to run; therefore, no information on the input data of the users is ever stored. Also, MPP is designed so that no login is required (Fig. 1).

To locally run the functionalities employed by MPP, the following dependencies are required: the Python modules SeqIO and ProteinAnalysis from the packages Bio and Bio.SeqUtils.ProtParam, respectively; the modules StringIO and BytesIO from the package io; and the re, csv, and matplotlib packages.

The application of MPP is hosted at a server located in Central Canada (Toronto) with the IP address 172.105.99.100. The Django-ready server runs the Debian 10 Linux distribution.

The deployment of MPP to its remote server, and consequently to the web for public use, is linked to a cloned Git repository, which is public at https://github.com/gustavsganzerla/mpp. A local version of GitHub (version 2.20.1) is installed at the remote server. Any subsequent changes to the MPP application are first locally implemented in a production server, then committed to the main Git repository, and finally pulled from the repository by the remote server.

### Supported Operations

MPP is able to read a collection of proteins as an uploaded .fasta file or manually input proteins in a textbox and return a list of computed properties of each protein. On its 1.0 iteration, MPP can perform the following tasks for each protein: *i*) determine the length; *ii*) calculate the GRAVY; *iii*) calculate the aliphatic index; *iv*) calculate the instability index; *v*) determine whether the protein is stable or unstable; *vi*) calculate the molecular weight; *vii*) calculate the aromaticity; *viii*) calculate the isoelectric point; *ix*) determine the charge at pH 7; *xi*) determine the fraction of secondary structure; *xii*) determine the molar extinction coefficient; xiii) calculate the amino acid composition and; *xiv*) calculate the atomic composition. Moreover, for properties *i* to *ix*, MPP can provide a visual plot encompassing all the input proteins. Below, we show the description of each property calculated by MPP.

i) Length: the total amino acids composing each input protein are calculated. An integer value is returned. Each protein is converted to a string and the *len()* function is applied. This property enables a plotting function, in which a histogram is provided for checking the frequency of the length of all input proteins.
ii) GRAVY: each amino acid composing an input protein has a hydropathicity value, previously calculated by Kyte and Doolittle [10], 1982 (the reference list is available at Supplementary Table S1); the GRAVY function of MPP sums the hydropathicity values of a protein and divides it by the length of the protein, thus returning a score. This property enables a plotting function, in which a scatter plot is provided for each individual GRAVY score calculated.
iii) Aliphatic index: calculates the aliphatic index of a protein sequence by first counting the occurrences of specific aliphatic (non-polar) amino acids (Alanine, Valine, Isoleucine, and Leucine), then computing their mole percentages within the protein. Using predefined constants ‘a’ and ‘b,’ it calculates the aliphatic index, which reflects the hydrophobicity of the protein. This index is based on the composition of these aliphatic amino acids and their contribution to the overall structure, with higher values indicating a higher aliphatic character in the protein sequence. The calculation was based on the finding of Ikai [11], (1980). This property enables a plotting function, in which a scatter plot is provided for each individual aliphatic index score calculated.
iv) Instability index: for calculating the instability index of input proteins, we used the Bio.SeqUtils.ProtParam module from BioPython. The function *instability_index(self)* calculates the stability of proteins. An input protein needs first to be read as a ProteinAnalysis object to be used as input parameter for the *instability_index(self)* function. This property enables a plotting function, in which a scatter plot is provided for each individual instability index score calculated. In addition, each plot generated by MPP’s calculation of the instability index will have a horizontal line in the instability value (y-axis) of 40. Proteins with the instability index above 40 indicate the protein is unstable and has a short half-life [12].
v) Stability: the threshold of 40 determines the stability of each protein. Values equal to or less than 40 are defined as stable while values above 40 are defined as unstable. This property enables a plotting function, in which a pie plot is generated accounting the proportion of stable and unstable proteins for a given input dataset.
vi) Molecular weight: for calculating the molecular weight of input proteins, we used the Bio.SeqUtils.ProtParam module from BioPython. The function *molecular_weight(self)* takes a protein read as a ProteinAnalysis object as input and returns the sum of the atomic weight of all atoms in the chemical structure of the protein. This value is represented in Daltons (Da). This property enables a plotting function, in which a scatter plot is provided for each individual molecular weight calculated.
vii) Aromaticity: for calculating the aromaticity of input proteins, we used the Bio.SeqUtils.ProtParam module from BioPython. The function *aromaticity(self)* takes a protein read as a ProteinAnalysis object as input and returns the relative frequency of aromatic amino acids (i.e., phenylanine, tryptophan, and tyrosine) that compose the input proteins. This property enables a plotting function, in which a scatter plot is provided for each individual aromaticity.
viii) Isoelectric point: for calculating the isoelectric point, we used the Bio.SeqUtils.ProtParam module from BioPython. The function *isoelectric_point(self)* takes a protein read as a ProteinAnalysis object as input and returns the pI of a protein, i.e., the pH in which the net charge of a protein molecule is zero. This property enables a plotting function, in which a scatter plot is provided for each individual pI.
ix) Secondary structure fraction: for calculating the fraction of the helix, turn, and sheet of a protein, we used the Bio.SeqUtils.ProtParam module from BioPython. The *secondary_structure_fraction(self)* function takes as input a protein read as a ProteinAnalysis object and returns the fraction of amino acids that tend to be in the helix, turn, and sheet of the protein. The amino acids valine (V), isoleucine (I), tyrosine (Y), phenylalanine (F), tryptophan (W), and leucine (L) are in the helix. The amino acids asparagine (N), proline (P), glycine (G), and serine (S) are in the turn. The amino acids glutamic acid (E), methionine (M), alanine (A), and leucine (L) are in the sheet as described in the documentation of ProtParam (https://biopython.org/docs/1.76/api/Bio.SeqUtils.ProtParam.html)
x) Molar extinction coefficient: for calculating the molar extinction coefficient of each input protein, we used the Bio.SeqUtils.ProtParam module from BioPython. The method *molar_extinction_coefficient(self)* returns the molar extinction coefficient of proteins containing cysteines and cystine (a dimer of cysteine residues connected by a disulfide bond).
xi) Atomic composition: for determining the atomic composition of the input protein, we accounted for the number of carbon (C), hydrogen (H), oxygen (O), nitrogen (N), and sulfur (S) atoms present in each amino acid composing each input protein. A chemical equation of the respective number of CHONS was returned. The number of CHONS molecules per each amino acid were taken from Sanvicto and Farci [3], (2020) and are available in Supplementary Table S2.

**Fig. 2:**
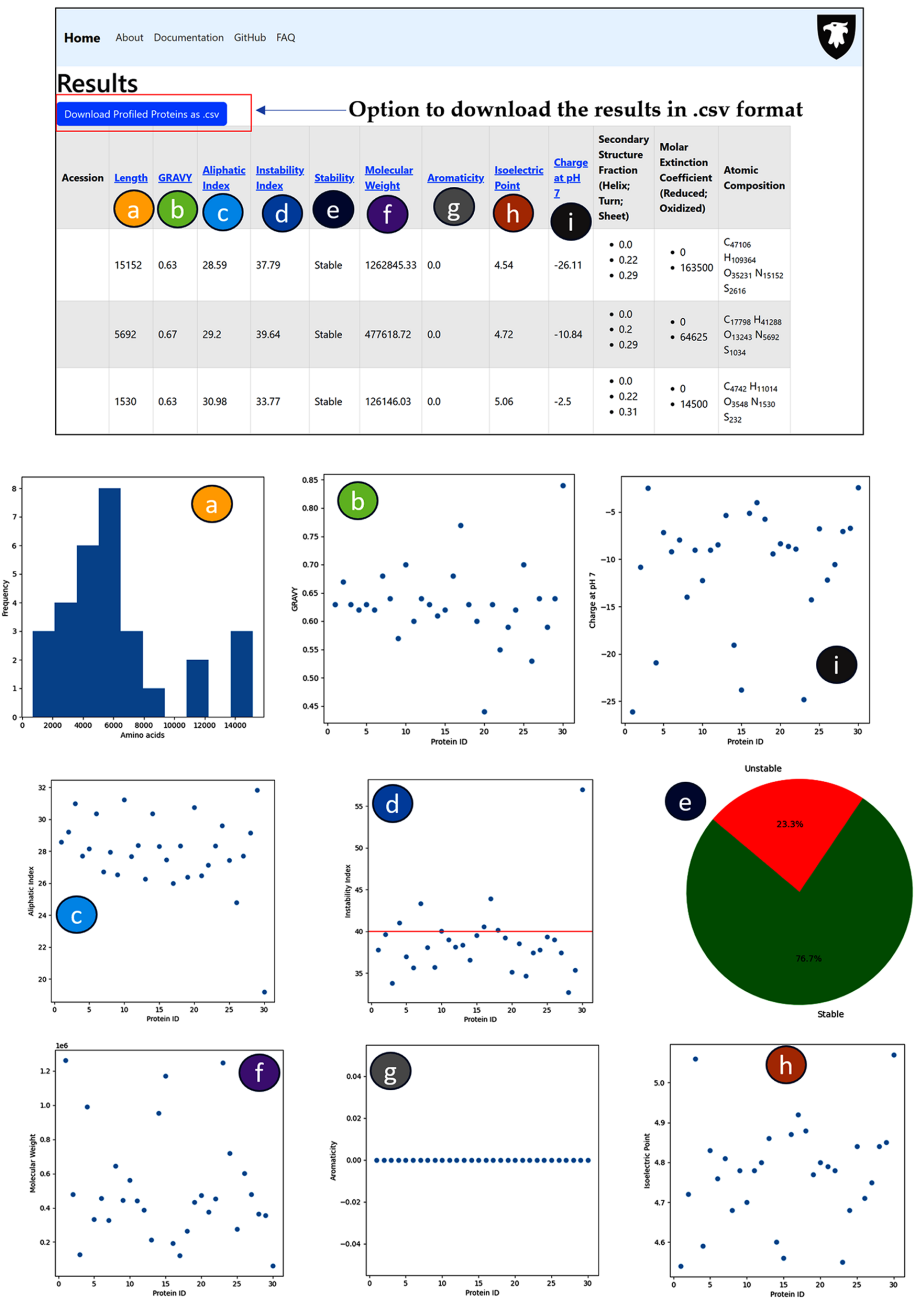
Graphical representation of MPP output for bulk sequences or whole proteome: (**a**) Lenth of amino acids; (**b**) GRAVY; (**c**) Aliphatic index; (**d**) Instability index; (**e**) Stability; (**f**) Molecular weight; (**g**) Aromaticity; (**h**) Isoelectric point; and (**i**) Charge at pH 7

Apart from the aforementioned calculation of properties of proteins, MPP also generates a downloadable .CSV file containing the accession number, protein sequence, protein description, length, GRAVY, aliphatic index, instability index, stability, molecular weight, aromaticity, isoelectric point, and charge at pH 7 of each input protein (Fig. 2).

Finally, MPP also enables the individual visualization of each input protein. In the results section, we included a URL in the accession number (first column) of each input protein that redirects the user to a subsequent page, displaying the accession number, description, amino acid sequence, length, GRAVY, aliphatic index, instability index, stability, molecular weight, charge at pH 7, atomic composition, and percentage of each amino acid of the selected protein. The generated plots are also downloadable as compressed (.zip) PNG files by clicking in the “Download Zipped Plots” button of the results page.

### Local Executable

Apart from its version running on a webserver, we also provide a version of MPP to be downloaded. In the page https://mproteinprofiler.microbiologyandimmunology.dal.ca/download, we include a downloadable compressed file containing (i) the MPP_local.py file and (ii) a readme file with instructions on how to execute. Interested users will need a command line environment with Python (version 3.12) installed. The command for executing MPP_local.py (i.e., python mpp_local.py < input.fasta > < output.csv> ) consists of two arguments; i.e., i) the input file containing the proteins in .fasta format, and the output file, preferably a .CSV. MPP_local will read the input file and calculate length, GRAVY, aliphatic index, instability index, molecular weight, aromaticity, isoelectric point, and charge a pH 7 of each protein and write the calculation results to the file specified in the second argument of MPP_local execution.

### Comparison with Other Tools

We provided a comparison of MPP and other tools for calculating physicochemical properties of proteins such as ProtParam [13], AAindex [14], and PeptideMass [15] (Table 1). In terms of the purpose of each tool, both ProtParam and MPP are designed to calculate various physicochemical properties while AAindex provides access to a database of aa indices and PeptideMass calculates the molecular weight of protein sequences. Regarding the tools functionalities, MPP differs from ProtParam as the second tool provides calculation of single proteins. While PeptideMass calculates monoisotopic and average mass of peptides, both MPP and ProtParam calculate the same properties such as molecular weight, theoretical pI, and aa composition, among others. In fact, some of the functionalities we applied at MPP are provided in the ProtParam Python module; however, while ProtParam is limited to profiling one protein at a time, it is not able to profile entire proteomes and/or lists of proteins in a single run. We acknowledge that the developers of ProtParam, by enabling their Python module, allowed users adapt the code to iteratively profile multiple proteins at once. However, Python programming proficiency on the user side would be required to do so. Finally, all the compared tools are available as webservices and standalone tools.

**Table 1:** Comparison between popular tools available for physicochemical properties calculations and newly developed MPP tool

Feature	ProtParam	AAindex	PeptideMass	MPP
Purpose	Calculates various physicochemical properties	Accesses a database of amino acid indices	Calculates the molecular weight of peptides	Calculates a set of physicochemical properties of the proteins or whole proteome
Functionality	Calculates various physicochemical properties	Accesses a database of amino acid indices	Calculates the molecular weight of peptides	Calculates a set of physicochemical properties of the proteins or whole proteome
Properties	Single protein sequence analysis	Database search for specific amino acid indices	Mass calculation for a sequence of amino acids	Single protein, bulk, or proteome-wide analysis
Input	Includes basic properties such as molecular weight, theoretical pI, amino acid composition, etc.	Provides access to a wide range of amino acid indices with diverse properties	Calculates the monoisotopic and average mass of a peptide	Basic properties such as molecular weight, theoretical pI, amino acid composition, etc.
Output	Single amino acid sequence	Amino acid index IDs or keywords for specific properties	Amino acid sequence	Amino acid sequences of single protein, bulk proteins, or whole proteome in fasta format
Availability	Computed physicochemical properties	Information on the selected amino acid indices	The molecular weight of the peptide	Option to download the computed physicochemical properties graphs and output table in .csv, .txt., and .xls as well as in a Zip folder

### Case study: Calculation of Physicochemical Properties of Mpox Virus (Formerly Known as Monkeypox Virus) Proteome using the MPP Tool

The whole proteome of the Mpox virus was downloaded from the UniProt repository (https://www.uniprot.org/proteomes/) in the fasta format with the accession number UP000516359. The download was found to contain 183 proteins, and all these proteins were simultaneously considered for the physicochemical properties using the newly developed MPP tool (https://mproteinprofiler.microbiologyandimmunology.dal.ca/). A set of physiochemical properties including molecular weight, isoelectric point, instability index, aliphatic index, and grand average of hydropathicity of whole proteome were computed.

Sequence analysis of the Mpox proteome revealed that the length of the proteins ranged from 42 (A0A7H0DNC9) to 1880 (A0A7H0DNG6) aa. The calculated molecular weight and isoelectric point ranged from 4905.9 (A0A7H0DNC9) to 212312.82 g/mol (A0A7H0DNC9), and from 4.05 (A0A7H0DN32) to 10.13 (A0A7H0DN00), respectively. Based on the physicochemical properties calculations, it was observed that out of 183 proteins, 103 (56.3%) were predicted as stable, while the remaining 80 (43.7%) were found to be unstable at the sequence level. As presented in Supplementary Table S3, the calculated instability ranged from 4.82 (A0A7H0DNB3) to 77.71 (A0A7H0DN25). The aliphatic index of Mpox proteins ranged from 34.84 (A0A7H0DN00) to 182.07 (A0A7H0DN68) with GRAVY score ranging from − 1.6 (A0A7H0DN25) to 1.5 (A0A7H0DNB3). These calculated scores indicated the flexible stability of TaGATA proteins at different ranges of temperatures. Two other important parameters (Aromaticity and Charge at pH 7) were also computed and noteworthy variations across the whole proteome were observed. The aromaticity of the Mpox virus proteins ranged from 0.01 (A0A7H0DNE1) to 0.19 (A0A7H0DNB3), whereas the pH 7 charge demonstrated a broader spectrum, ranging from − 30.24 (Q8BEJ6) to 16.7 (A0A7H0DNC3). The results of the calculated physicochemical properties by using the MPP tool are graphically plotted in Supplementary Figures S4, S5, S6, S7, S8, S9, S10, S11 and S12. In conclusion, the calculated physicochemical properties of the Mpox virus proteome may help to select the candidate target proteins based on the physicochemical profiling for accelerating the epitope mapping and screening of potential inhibitors against contagious Mpox disease.

## Concluding Remarks

In developing MPP, we aimed to provide a webtool that allows users to input entire proteomes or custom-made lists of proteins for profiling by our tool. The development philosophy we applied for building MPP allows us to iteratively add new features to our tool. Finally, the transparent development of MPP, for which the source code is publicly available in a repository, welcomes interested users and developers to contribute to the lifecycle of MPP.

### Electronic Supplementary Material

Below is the link to the electronic supplementary material.

Supplementary Material 1

Supplementary Material 2

Supplementary Material 3

## Data Availability

No datasets were generated or analysed during the current study.
